# Automating Racism: Is Use of the Vaginal Birth After Cesarean Calculator Associated with Inequity in Perinatal Service Delivery?

**DOI:** 10.1007/s40615-024-02233-4

**Published:** 2024-12-14

**Authors:** Nicholas Rubashkin, Ifeyinwa V. Asiodu, Saraswathi Vedam, Carolyn Sufrin, Miriam Kuppermann, Vincanne Adams

**Affiliations:** 1 Department of Obstetrics, & Reproductive Sciences, University of California at San Francisco, 490 Illinois St, GynecologySan Francisco, CA 1025594158, USA; 2 Institute for Global Health Sciences, University of California at San Francisco, San Francisco, CA, USA; 3 Department of Family Health Care Nursing, School of Nursing, University of California, San Francisco, USA; 4 Birth Place Lab, Faculty of Medicine, University of British Columbia, Vancouver, Canada; 5 School of Population & Public Health, Faculty of Medicine, The University of British Columbia, Vancouver, Canada; 6 Department of Gynecology & Obstetrics, Johns Hopkins University School of Medicine, Baltimore, MD, USA; 7 Department of Anthropology, History and Social Medicine, University of California, San Francisco, USA

**Keywords:** Algorithms, Cesarean birth, Racism, Health equity

## Abstract

**Objective:**

The clinical application of race-adjusted algorithms may perpetuate health inequities. We assessed the impact of the vaginal birth after cesarean (VBAC) calculator, which was revised in 2021 to address concerns about equity. The original algorithm factored race and ethnicity and gave lower VBAC probabilities to Black and Hispanic patients.

**Methods:**

From 2019 to 2020, we conducted a multi-site, ethnographic study consisting of interviews and audio recordings of 14 prenatal visits. We used grounded theory to describe the social processes of racialization.

**Findings.:**

Across 4 sites, 12 obstetricians, 5 midwives, and 31 pregnant/postpartum patients participated. Seventy-four percent (*N* = 23) of the pregnant/postpartum individuals identified as racially minoritized, and the remaining 24% (*N* = 8) identified as White. We identified four processes that facilitated the “automation” of racism: adhering to strict cutoffs; the routine adoption of calculators; obfuscating the calculator; and the reflexive categorization of race and ethnicity. When clinicians adhered to strict cutoffs, they steered low-scoring Black and Hispanic patients toward repeat cesareans. If clinicians obfuscated the calculator, Black and Hispanic patients had to work to decode the role of race and ethnicity in their probabilities in order to pursue a VBAC. By reflexively categorizing race and ethnicity, the use of the calculator forced patients to choose a singular identity, even if it obscured the truth about their multi-faceted race or ethnicity.

**Conclusion:**

The VBAC calculator’s inclusion of race and ethnicity helped to automate racism by coding race into institutional practices and care interactions. This resulted in some clinicians discouraging or prohibiting Black and Hispanic patients from attempting a VBAC.

**Significance:**

To date, no empiric study has examined whether the VBAC calculator produced inequities in access to VBAC services and reproduced racism in care. The VBAC calculator resulted in fewer VBAC attempts among racially minoritized patients, denying them the opportunity to undergo labor and a vaginal birthing experience.

## Objective

In perinatal care, developing an algorithm to assess a patient’s specific probability for having a successful vaginal birth after cesarean (VBAC) could be more informative to person-centered decision making conversations than the standard approach of quoting patients a range of successful VBAC rates between 60 and 90% [1]. Scheduling a repeat cesarean or attempting a VBAC may both be safe and reasonable options, and as a result, patient preferences play an essential role in deciding an approach to birth after a cesarean [2]. Since patients assign different meanings to different numeric probabilities for having a VBAC [3], it is useful to scrutinize how these decisions are made.

**Fig. 1:**
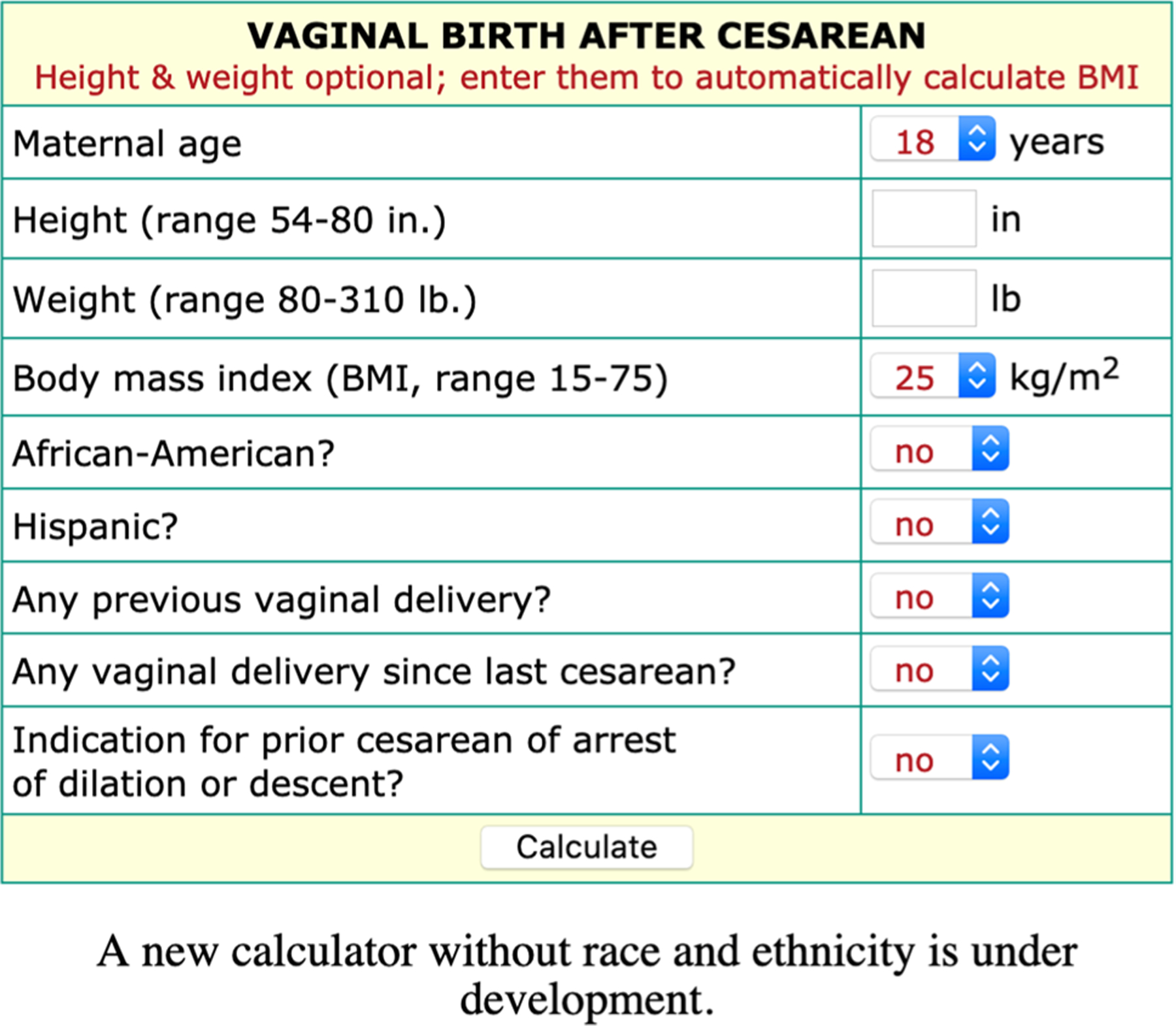
The MFMU VBAC calculator as it appeared on a NIH-hosted website from the early 2010s until it was revised and taken down in 2021. Note: the inputs pictured here (e.g., 18 years, BMI of 25) represented the calculator’s default settings

In 2007, the Maternal Fetal Medicine Units (MFMU) Network created a VBAC algorithm that incorporated race and ethnicity as one of several variables along with age, body mass index (BMI), and the indication for the prior cesarean [4] (see Fig. 1). The MFMU algorithm revealed that patients who attempted a VBAC with scores below 60% experienced twice the morbidity compared to those who had similarly low scores but scheduled a repeat cesarean [5]. In theory, the MFMU model could help women avoid risk by scheduling a cesarean when scores drop below 60%. Due in large part to this evidence base, the MFMU’s algorithm, which became known as *the* VBAC calculator, was widely adopted in the U.S. [6] and was tested in several international validation studies [7, 8].

More than a decade after its adoption, skepticism and concern around the VBAC calculator’s use of race and ethnicity surfaced [9, 10]. Because of the way the MFMU algorithm factored race and ethnicity, it assessed Black and Hispanic patients as having probabilities that were on average 5–15 percentage points lower than White women with identical clinical factors [4]. Scholars began to critique race-adjusted clinical algorithms more broadly for perpetuating a biological construct of race [11], as well as for obscuring the structural root causes of health disparities, such as racism [12]. Specifically, the concern was that the utilization of race-adjusted algorithms in clinical care would perpetuate historical structural inequities [13, 14]. We are unaware of any empiric studies that have examined whether the VBAC calculator perpetuated inequities in access to VBAC and reproduced racism in care. In this study, we used ethnographic methods to describe social processes of racialization involved with the real-time utilization of the VBAC calculator by clinicians and patients. We analyzed how these processes might contribute to the reproduction of the calculator’s inequitable assessment of Black and Hispanic patients as it relates to accessing VBAC services. We also sought to identify ways in which clinicians and patients might disrupt the reproduction of racism that, we argue, was “automated” by the calculator.

## Methods

### Design

From April 2019 to October 2020, we conducted a comprehensive ethnographic study at geographically diverse sites. For this paper, we analyzed a subset of data provided by patients and clinicians. Approval to conduct this study was received from the University of California, San Francisco Institutional Review Board.

### Setting and Sample

We recruited clinicians and patients from 4 sites: 1 academic hospital in the Northeast, 1 community practice in the Southwest, and 2 academic hospitals in Northern California. In the Northeast and Southwest, we recruited postpartum participants through social media and snowball sampling. In Northern California, we recruited clinicians via email using a short practice survey to identify those with experience using the calculator. Research staff identified eligible pregnant participants using the Electronic Health Record. Then, we purposively sampled participants to obtain a range of calculator scores. To participate, patients had to be pregnant or postpartum, have had a prior cesarean, speak English or Spanish, and be over the age of 18.

### Data Collection

After verbal informed consent, we gathered demographic and professional information from clinicians and then conducted a single semi-structured interview. After written informed consent, we gathered basic demographic and clinical information from pregnant/postpartum patients. All participants self-identified their race and ethnicity. Pregnant/postpartum participants could then opt into a series of interviews and audio recordings of their prenatal visits. Prenatal visits were recorded via a digital audio recorder in the exam room. Participants who did not encounter the calculator during a prenatal visit were presented with their scores in a follow-up interview. As the study progressed interview guides were honed to focus on emergent findings. The interviews were completed in person, over the phone, or via Zoom. Participants were reimbursed $25–50 depending on the length of the interview and $50 for visit recordings. See Table 1 for sample questions from the interview guides.

### Analysis

We analyzed the data thematically in Atlast.ti using a critical race lens applied to algorithmic racism [15]. Thus, our analysis sought to identify likely social and structural processes through which the VBAC calculator could encode racism. We analyzed how the calculator’s design propagated inequity through institutional practices, information technology systems, and care interactions—what we call “automation.” Wherever the VBAC calculator became embedded, we explored automation by considering how a given process facilitated the calculator’s inequitable assessment. By contrast, we also considered how in any given process the calculator’s assessment could be disrupted. Using an ethnographic approach to grounded theory [16, 17], data collection and analysis happened concurrently, and data collected later in the study was tested against preliminary themes. Over the course of the study, as themes solidified, we pursued “theoretical sampling” to test identified themes against additional data [18]. Data saturation was reached when no new information emerged with additional recruitment.

In presenting the results, we describe clinicians by their race/ethnicity and professional role. We withheld provider gender as this could potentially make some clinicians identifiable. We identify pregnant and postpartum participants using a pseudonym with their racial/ethnic self-identification and calculator score to help contextualize the results.

## Results

**Fig. 2:**
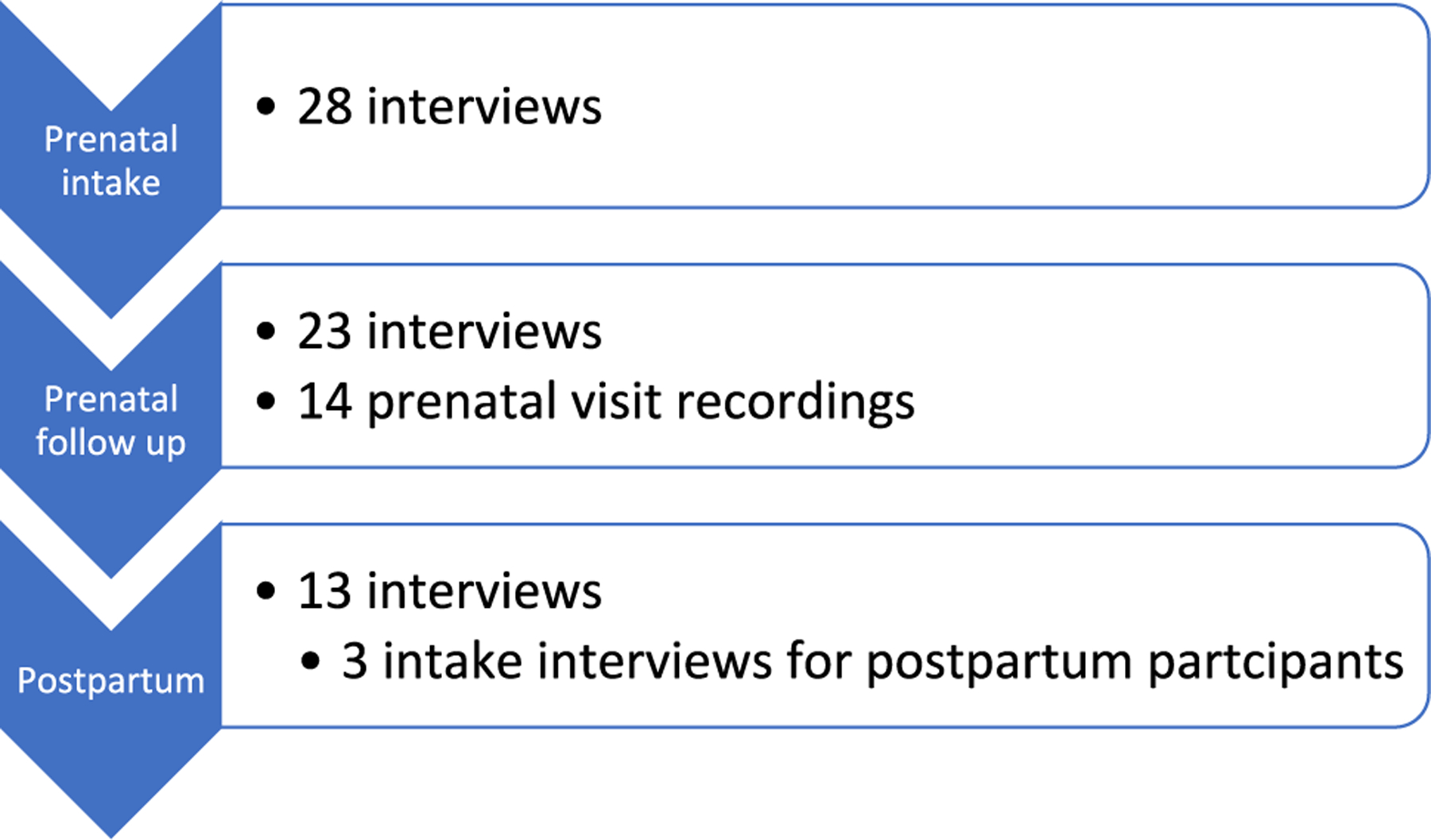
Pregnant/postpartum participant data collection (*N* = 64 interviews, *N* = 14 recordings)

In total, 17 clinicians (Table 2) and 31 pregnant/postpartum patients (Table 3) enrolled in the study. Patients participated in 78 data collection events (64 interviews; 14 prenatal visit recordings) with an average of 2.5 events per participant and a range of 1–6 (see Fig. 2). The analysis surfaced four processes that facilitated the automation of inequities via the calculator’s assessment: *adhering to strict cutoffs*; *the routine adoption of calculators*; *obfuscating the calculator*; and *reflexively categorizing race and ethnicity*.

### Finding 1: Adhering to Strict Cutoffs

Clinicians reported varying degrees of and rationales for using cutoff scores to determine who could be offered a VBAC. At one site, a provider reported, “The policy was you calculate, you get their score and if it’s less than 63%, you do a C-section. If it’s more than 63% percent, you can offer them a VBAC” (White general Ob/Gyn). Some clinicians struggled with such policies and argued that the cutoff score should not supersede a patient’s preference for a VBAC. Other clinicians believed the cutoffs helped to keep patients safe, basing this belief on the MFMU’s determination of greater morbidity in attempted VBACs in the setting of scores below 60%.

One site that used cutoff scores designed a quality improvement (QI) project to identify clinicians who might be inappropriately supporting low-scoring women to pursue VBACs. The QI project adjusted the EHR to require that clinicians, to complete their charting, categorize their patients according to quartiles of calculator scores. Partitioning patients into quartiles allowed the QI project to assess the distribution of VBAC calculator scores. A provider involved in the project hypothesized, “If they [VBAC candidates] are evenly distributed, well then wouldn’t that suggest that the calculator wasn’t helping?” (White General Ob/Gyn).

Despite the QI project’s enforcement of cutoffs, a provider could, to some extent, exercise judgement and support a low-scoring patient who wanted a VBAC. One such Ob/Gyn extensively counseled a patient named Destiny (Black, score 12%) about her birth options. When Destiny arrived at the hospital for an induction of labor, the on-call Ob/Gyn consulted with Destiny’s primary Ob/Gyn. “The colleague contacted me that she was going to go along with the plan, however, she did not think that the patient had been appropriately counseled” (White General Ob/Gyn). Destiny’s Ob/Gyn affirmed that Destiny had been well counseled and advocated for the agreed-upon birth plan. Unaware of the behind-the-scenes controversy, Destiny achieved the VBAC that she had so desired.

### Finding 2: The Routine Adoption of Calculators

Even in institutions that did not require the use of the calculator, soon after its publication, the calculator was adopted into routine counseling at many sites. The VBAC calculator became a standard for educating Ob/Gyn trainees, who demonstrated their competence by conducting individualized risk assessments. “So we were required as a part of residency to always put those numbers [from the calculator] in…Then the attending would want to see what their percentage was” (White MFM). Ob/Gyn trainees also integrated the calculator into their “chart prepping” activities to make the visit more efficient. “I feel like as a resident, it was part of our checklist …so we would calculate the chance of success for every single patient” (Black MFM).

Established clinicians adopted the calculator because they knew the MFMU to be reputable and because the ACOG guidelines discussed the calculator. Clinicians often referred to their use of the calculator as evidence-based to justify its integration into routine clinical practice. When one clinician introduced the calculator, the adoption radiated out to colleagues. In this way, the calculator standardized the counseling approach in large group practices. As one clinician referred to her colleague who introduced both the calculator and cutoffs: “She’s super evidence-based, and she said, hey guys, because we’re such a shared practice, we share everything antenatally and we share call, let’s come up with a line in the sand [cutoffs] to be consistent across the group” (White general Ob/Gyn). In the absence of using cutoff scores, some clinicians reported using the calculator to launch into a broader informed decision making discussion about risks and benefits. Other clinicians stopped using the calculator, observing that the score did not matter for patients, especially those who already had a strong preference for VBAC or repeat cesarean.

As critiques around the inclusion of race in epidemiologic models publicly proliferated, some clinicians began to reevaluate the routine adoption of the calculator. One such provider, an MFM who identified as Latinx, recalled being socialized into using the calculator during their training. “I feel like I was indoctrinated to the calculator…It’s like this script that we’ve passed on but at no point were we really talking about why are each of these steps so important.” The MFM struggled with the calculator’s stigmatization of Hispanic women. “It’s like we [Latinas] can’t win. If there’s a negative connotation to having multiple pregnancies or multiple vaginal [births]…it’s going to be coming from a Latina.” As clinicians reevaluated their use of the calculator, some began to exclude race and ethnicity from their calculations, by categorizing everyone as White, or they stopped using the calculator entirely.

### Finding 3: Obfuscating the Calculator

In practice, despite the belief they were being guided by objective evidence, clinicians often subjectively interpreted the calculator’s probability as more or less favorable for the patient. Some clinicians volunteered such interpretations for patients. Other times, patients requested that clinicians help interpret the score. As clinicians gained facility with the calculator, some rendered their interpretations without explicitly referencing the calculator. When clinicians obfuscated either the existence of the calculator or how it worked, they made it more likely that the calculator’s unequal assessment would remain hidden from the patient.

Clinicians often interpreted high scores using encouraging phrases that carried statistical weight. At a prenatal visit, Justine’s (White, 77%) MFM presented her calculator score in a positive light. “I kind of plugged you into the calculator and it’s predicting a success rate in the 70s…So pretty much your factors are in your favor” (Prenatal visit recording). By saying that Justine’s risk factors were in her favor, the MFM was referring to Justine having had a prior cesarean for breech, but the role of Whiteness in the score was folded into the positive interpretation. Despite having risk factors that supported a successful VBAC, Justine still had the option to schedule a cesarean, which is what she did. Women with high scores had unrestricted choice, and as a result, women like Justine did not have to push her provider further to explain why her risk factors supported a successful VBAC.

On the other hand, Black, Hispanic, and some multiracial and multi-ethnic women encountered discouraging interpretations of their scores from clinicians. In her first prenatal visit, Chloe (Black, 25%), who had a cesarean when her labor stopped progressing, queried her Ob/Gyn about whether VBAC might be an option for her. Chloe recalled that the Ob/Gyn reacted decisively, initially without a reference to the calculator. “She [the Ob/Gyn] was like, ‘Oh no. We’ll schedule your C-section.’ She didn’t even ask me questions. So that was very frustrating. I asked her, ‘Okay, tell me more about why—’ It felt like I was pulling from her some of these factors.” Chloe had to perform extra work to expose the calculator, and the Ob/Gyn eventually explained, “‘Well your weight, you’re African-American, you’re 35’… She did lay out the factors.”

### Finding 4: Reflexively Categorizing Race and Ethnicity

The calculator required clinicians to categorize patients into mutually exclusive racial and ethnic categories of Black, Hispanic, and White. When the patient’s self-reported racial and ethnic identities aligned with the calculator’s structure, categorizing went smoothly. Other times, clinicians encountered challenges with entering Asian-Americans and Indigenous people, multi-racial and multi-ethnic patients, or Latinx people of African descent. Sometimes these challenges were insurmountable and a provider declined to use the calculator in that particular instance. Other times, when confronted, for example, with a hypothetical patient who reported mixed-African ancestry, a provider reported that they would reflexively apply a “one drop” rule: “We probably say they’re African-American if they’re any part” (White General Ob/Gyn).

As someone who had a Hispanic mother and a White father, Beatriz (mixed-Hispanic and White, 40% or 56%) offers an example of how reflexively categorizing influenced scoring. Beatriz often passed as White and indicated her ethnicity as White in other official documents. In the context of her prenatal care, Beatriz understood Hispanic women to be “at risk” for certain conditions like diabetes, and as a result, she volunteered her Hispanic heritage so that her clinicians could have complete data about her health. While discussing the calculator in a visit, Beatriz’s Ob/Gyn encouraged her to identify as White. “The doctor said, ‘Are you sure? Because you look White.’ I was like, ‘Yes, I’m sure.’ She’s like, ‘Okay, I’m just saying this because if I mark Hispanic, it’s going to knock you down a few points.’” Beatriz was giving birth at a site that used cutoffs, so the exact numeric score mattered if she were to try a VBAC. That the calculator could have such disparate results based on the visual perception of her ethnicity revealed to Beatriz the arbitrariness between looking White and being Hispanic. “I was infuriated at that time…I was just thinking like, this just shows how arbitrary this entire process is. I’m like, if this calculator is based on science, then it shouldn’t be like that flexible.”

## Conclusions for Practice

While some have expressed concern that the VBAC calculator might systematically disadvantage Black and Hispanic patients [9], our qualitative study clearly documents how that disadvantage is operationalized even when racial bias is a known concern. By naturalizing incorrect biological notions of racial and ethnic differences in relation to greater “failure” rates among Black and Hispanic patients, the science and practice of the VBAC calculator contributed to obstetric racism in the United States [19, 20]. While the calculator assessed Hispanic women as less able to have successful VBACs, as pointed out in our study, Hispanic women are also paradoxically racialized as “obstetrically hardy [21, 22].” In both instances, racism, not race, produces Hispanic women as at risk compared to normal White bodies.

Increased awareness around the role of racism in producing inferior outcomes for Black birthing people and their newborns [23, 24] coincided with the obstetric profession adopting an evidence-based yet discriminatory technology. As others have shown, algorithms have the potential to inscribe historical racism into new information architectures, institutional practices, and human interactions [13]. We call this the “automation” of racial bias through medical/diagnostic technologies.

When clinicians used cutoff scores, they facilitated the calculator’s inequitable assessment. In a national survey of Certified Nurse Midwives (CNMs), 40% reported that their collaborating physicians required the use of the calculator, and another 21% reported using cutoff scores to prohibit or discourage VBAC [25]. When clinicians did not transparently demonstrate the calculator in their counseling, Black and Hispanic patients had to perform extra work to expose the calculator’s inequitable assessment. Adhering to cutoff scores and obscuring the calculator both fit into a pattern of what Altman and colleagues have called “information packaging,” a process in which prenatal clinicians selectively share vital information from pregnant people of color about their care [26].

While there may be a greater relative risk of complications when attempting a VBAC with scores below 60% [27], this finding may need to be verified with a race-neutral VBAC prediction model. Furthermore, bioethicists and professional guidelines advise against using relative risk to restrict access to VBAC [2, 28, 29], given the overall small absolute risks and the preference-sensitive nature of decisions in this context. Indeed, some providers went against official policy to support low-scoring patients in their plans for a VBAC. Furthermore, because the calculator sometimes focused counseling around a limited set of risk factors, other factors that influence VBAC success, such as patient preference and the benefits of midwifery care, were excluded from informed choice discussions [3, 30].

The calculator’s structure required that providers reflexively categorize patients into mutually exclusive racial and ethnic groups, demonstrating again the powerful and political role of epidemiology in redefining racial differences as quantifiable and objective [31, 32]. As revealed by patients in this study who did not neatly fit into the calculator’s categories, the calculator was powerful precisely because it appeared to be scientifically designed to ensure a safe birth but promoted the false idea that it is possible to biologically divide humanity into mutually exclusive races, via “one-drop” rules and the like. This practice reinforced racism. As the public conversation around “racism not race” grew, some providers began to reflexively exclude race and ethnicity from their calculations due to a new perception of the calculator’s flawed design.

### Strengths and Limitations

A strength of our study was its longitudinal design that allowed for in-depth explorations as pregnant participants ran into the calculator in real time. We also note several limitations. We enrolled a limited number of Black and Indigenous participants. Also, we gathered most of our data from the two Northern California sites, both of which did not utilize cutoff scores.

## Conclusion

In 2021, amidst growing calls for the abolition of race-based medicine [11], the MFMU developed a new calculator that excludes race and ethnicity as a variable [33]. While removing race and ethnicity will help mitigate the VBAC calculator’s most negative consequences and may not affect prediction accuracy [34], racism might continue to operate implicitly. Racism may explain in part why Black and Hispanic pregnant people undergo more unnecessary primary cesareans [35]. Because the new VBAC calculator treats every prior cesarean as if it were clinically necessary, more Black and Hispanic pregnant people become implicitly eligible for entry into a VBAC prediction tool. If a fairer and more just VBAC calculator exists, it would have to pay attention to the explicit and implicit ways that racism structures the risk of primary and repeat cesareans. Otherwise, the possibility remains that a VBAC prediction model could perpetuate historical structural racism [36].

## Figures and Tables

**Table 1:** Sample interview guide questions for clinician and pregnant/postpartum participants

Providers	
General approach to counseling patients about repeat cesarean and VBAC	Can you describe your approach to counseling patients who have had a prior cesarean? How has your approach to counseling changed over time?
Utilization of the calculator	How did you decide to use/not use the calculator? How were you trained to use the calculator? How do you use the calculator now? Can you describe a recent case where you found the calculator to be helpful or challenging to use? How does your practice/hospital approach the calculator? What are your future plans for the calculator?
Pregnant/postpartum patients	
Understanding the context and factors influencing birth after cesarean	Tell me more about what happened leading up to and during your cesarean? How did this first cesarean affect you? How did you learn about VBAC/repeat cesarean as an option for your next pregnancy? What/who have been helpful sources of information about these options?
Current pregnancy	How are your visits going so far? How has the topic of attempting a VBAC or scheduling a repeat cesarean come up? How did that conversation go? Based on your prior birth experience, what is important to you in this pregnancy?
Utilization of the calculator	How did the VBAC calculator come up during your visit? What was your reaction to the score? What did you find helpful/unhelpful about the score you were given? What are your thoughts on why race and ethnicity are included in the calculation?
Postpartum	What was your VBAC/repeat cesarean like for you? How do you feel about your decision for VBAC/repeat cesarean?

**Table 2:** Provider demographic and professional characteristics

Age	41.2 (31–66)
Gender identity	17 female
Profession	5 maternal–fetal medicine 2 Ob/Gyn residents 4 certified nurse midwives 5 general Ob/Gyn 1 certified professional midwife
Race and/or ethnicity	1 Asian/South Asian 2 Black 3 Hispanic 11 White
Geographic location of current practice	13 Northern California 1 Southwest 3 Northeast

**Table 3:** Pregnant/postpartum participant demographic and clinical characteristics

Age	34.2 (25–41)
Enrolled while currently pregnant	28
Enrolled when already postpartum	3
Prior VBAC/vaginal birth	6
Geographic location	28 Northern California 2 Southwest 1 Northeast
Average calculator score (range)	57.5% (12–95%)
Race/ethnicity	2 Asian/South Asian 4 Black 12 Hispanic (3 Spanish speakers) 1 Indigenous 8 White 1 mixed-Black/Asian 1 mixed-White/Asian 2 mixed-White/Hispanic
Birth outcome for pregnancy after first cesarean	13 VBAC 10 unplanned cesareans after labor started 8 elective repeat cesareans

## Data Availability

The raw interview data is available upon request.

## Code Availability

The data were coded in Atlas.ti, and the files are available upon request.
